# Providing male rats deficient in iron and n-3 fatty acids with iron and alpha-linolenic acid alone affects brain serotonin and cognition differently from combined provision

**DOI:** 10.1186/1476-511X-13-97

**Published:** 2014-06-13

**Authors:** Jeannine Baumgartner, Cornelius M Smuts, Michael B Zimmermann

**Affiliations:** 1 Centre of Excellence for Nutrition, North-West University, Private Bag X6001, 2520 Potchefstroom, South Africa; 2 Laboratory of Human Nutrition, Institute of Food, Nutrition and Health, ETH Zürich, Zürich, Switzerland

**Keywords:** Alpha-linolenic acid, Cognition, Iron, Monoamines, n-3 fatty acids

## Abstract

**Background:**

We recently showed that a combined deficiency of iron (ID) and n-3 fatty acids (n-3 FAD) in rats disrupts brain monoamine metabolism and produces greater memory deficits than ID or n-3 FAD alone. Providing these double-deficient rats with either iron (Fe) or preformed docosahexaenoic acid (DHA)/eicosapentaenoic acid (EPA) alone affected brain monoamine pathways differently from combined repletion and even exacerbated cognitive deficits associated with double-deficiency. Iron is a co-factor of the enzymes responsible for the conversion of alpha-linolenic acid (ALA) to EPA and DHA, thus, the provision of ALA with Fe might be more effective in restoring brain EPA and DHA and improving cognition in double-deficient rats than ALA alone.

**Methods:**

In this study we examined whether providing double-deficient rats with ALA and Fe, alone or in combination, can correct deficits in monoamine metabolism and cognition associated with double-deficiency. Using a 2 × 2 design, male rats with concurrent ID and n-3 FAD were fed an Fe + ALA, Fe + n-3 FAD, ID + ALA, or ID + n-3 FAD diet for 5 weeks (postnatal day 56–91). Biochemical measures, and spatial working and reference memory (using the Morris water maze) were compared to age-matched controls.

**Results:**

In the hippocampus, we found a significant Fe × ALA interaction on DHA: Compared to the group receiving ALA alone, DHA was significantly higher in the Fe + ALA group. In the brain, we found significant antagonistic Fe × ALA interactions on serotonin concentrations. Provision of ALA alone impaired working memory compared with age-matched controls, while in the reference memory task ALA provided with Fe significantly improved performance.

**Conclusion:**

These results indicate that providing either iron or ALA alone to double-deficient rats affects serotonin pathways and cognitive performance differently from combined provision. This may be partly explained by the enhancing effect of Fe on the conversion of ALA to EPA and DHA.

## Background

Children and adolescents from low-income countries may suffer from both, iron (Fe) deficiency (ID) and n-3 fatty acid deficiency (n-3 FAD) due to poor quality diets during periods of rapid growth [1,2]. Fe is a co-factor for enzymes involved in cell division and the synthesis of neurotransmitters, myelin and biologically active lipid mediators and is therefore crucial for normal brain development and functioning [3-7]. Similarly to Fe, the long-chain polyunsaturated n-3 FA docosahexaenoic acid (DHA, 22:6n-3) and eicosapentaenoic acid (EPA, 20:5n-3) play important roles in neuronal growth and differentiation, mainly by modulating physical properties of biological membranes and gene expression of various proteins, as well as in myelination and neurotransmission [8-11]. Therefore, a combined ID and n-3 FAD occurring early in life may exert more severe effects on brain development and functioning than an ID or n-3 FAD alone.

We have recently shown in male rats that ID and n-3 FAD in combination impairs monoaminergic neurotransmission and reference memory in the Morris water maze (MWM) to a greater extent than ID or n-3 FAD alone [12]. In a subsequent study we repleted double-deficient rats with Fe and a mixture of DHA and EPA, alone and in combination, for a period of 5 weeks. We found that the repletion with either Fe or DHA/EPA alone affected monoamine pathways differently from the combined repletion and, surprisingly, even exacerbated the working memory deficits associated with double deficiency [13].

Approximately 35% of brain lipids occur in the form of polyunsaturated FA, with arachidonic acid (ARA; 20:4n-6) and DHA being the main contributors [14]. The essential n-3 FA, alpha-linolenic acid (ALA), makes up less than 1% of the brain’s lipids and thus is not considered to play a direct role in brain health [15]. Indirectly, however, the intake of ALA may be crucial for brain development and functioning via its conversion to EPA and DHA. This might be particularly the case in human populations who do not consume fish and who do not have access to, or cannot afford fish oil supplements. In these populations improving DHA and EPA status by increasing the consumption of ALA-rich oils (e.g. flaxseed, canola, and soybean oils) might be a more suitable approach. However, tracer studies have shown that the rate of conversion of ALA to DHA is low in humans (1 - 5%) [16-18], but has been reported to be efficient in rats [19,20]. A recent study in rats showed that by decreasing linoleic acid (LA; 18:2n-6) in the background diet and by keeping the total dietary polyunsaturated fatty acid (PUFA) intake low it is possible to enhance the DHA status of rats fed diets containing only ALA as a source of n-3 FA [21]. However, it is not yet known whether this finding can be extended to the human situation. Furthermore, Fe is a co-factor of the elongase and desaturase enzymes responsible for the conversion of ALA to EPA and DHA [17]. Several previous rodent studies have found alterations in blood and brain FA composition as a consequence of ID [22-25].

Thus, the provision of ALA in combination with Fe might be more effective in repleting brain EPA and DHA concentrations, and in reversing the deficits associated with n-3 FAD, than the provision of ALA alone, particularly in ID subjects. Therefore, the aim of this study was to investigate whether repleting double-deficient rats with ALA and Fe, alone or in combination, can correct deficits in brain monoamine metabolism and cognition associated with deficiency.

## Results

### Brain weight, food intake, and body weight gain

At postnatal day (PND) 92, the body weight of ID + n-3 FAD rats was significantly lower than of age-matched controls (*p* <0.001) (Table 1). Fe repletion significantly increased total body weight gain (p <0.001) compared to the rats receiving an ID diet and compared to controls. Furthermore, compared to the rats that received an ID diet, Fe repletion significantly increased food intake to the level of control rats. However, body weight at PND 92 remained lower in the experimental groups compared to the control group (*p* <0.05, all experimental groups vs. control).

**Table 1:** Weight gain, food intake, and brain weight of male control rats and ID + n-3 FAD rats repleted with an Fe + ALA, ID + ALA, Fe + n-3 FAD, or ID + n-3 FAD diet for 5 wk

						***p*** **Value**		
	**Control**	**Fe + ALA**	**ID + ALA**	**Fe + n-3 FAD**	**ID + n-3 FAD**	**Fe**	**ALA**	**Fe × ALA**
Body weight, *g*	409 ± 8.0	338 ± 12.5*^,Δ,¥^	302 ± 7.4*^,#^	320 ± 7.8*^,¥^	290 ± 7.1*^,#,‡^	0.001	0.29	0.83
Total body weight gain, *g/35d*	124 ± 4.4	167 ± 9.4*^,Δ,¥^	136 ± 3.8^#,‡^	154 ± 4.0*^,Δ,¥^	138 ± 4.3^#,‡^	0.001	0.43	0.30
Total food intake, *g/35d*	711 ± 14.3	701 ± 26.1^Δ,¥^	624 ± 12.1*^,#,‡^	666 ± 12.2^Δ,¥^	602 ± 9.0*^,#,‡^	<0.001	0.14	0.78
Relative weight gain, *g/g food intake*	0.17 ± 0.01	0.24 ± 0.01*^,Δ^	0.22 ± 0.01*^,#^	0.23 ± 0.00*	0.23 ± 0.00*	0.20	0.98	0.14

Values are means ± SEM, *n* = 8–10 per group. *Abbreviations*: ALA, alpha-linolenic acid; n-3 FAD, n-3 fatty acid deficient; Fe, iron; ID, iron deficient.

*p* Values correspond to two-way ANOVA to test effects of dietary Fe (deficient vs. sufficient) and dietary ALA (deficient vs. sufficient), and Fe × ALA interactions.

*P <0.05 versus control group, analyzed using 1-way ANOVA with diet as between-subject variable followed by post hoc Dunnett’s test to determine whether experimental (repletion) groups differed from age-matched positive-control rats after repletion with Fe and/or ALA.

^#^*P* <0.05 versus Fe + ALA group; ^Δ^*P* <0.05 versus ID + ALA group; ^‡^*P* <0.05 versus Fe + n-3 FAD group; ^¥^P <0.05 versus ID + n-3 FAD group; Analyzed using 1-way ANOVA with diet (excluding control group) as between-subject variable followed by post hoc Tukey’s test to determine differences between experimental (repletion) groups.

### Brain Fe

Brain Fe concentrations in all brain regions were significantly lower in the ID + n-3 FAD rats than in the age-matched controls at PND 92 (*p* <0.05) (Table 2). After repletion, there was a significant effect of Fe for higher Fe concentrations only in the olfactory bulb (OB). In the OB, there was further an effect of ALA for higher Fe concentrations. The Fe + ALA group had significantly higher Fe concentrations in OB than the Fe + n-3 FAD (*p* = 0.023), the ID + n-3 FAD (*p* = 0.010) and the ID + ALA (*p* = 0.031) groups. The Fe concentrations in the OB of Fe + n-3 FAD and ID + n-3 FAD rats remained significantly lower than of age-matched controls. In the frontal cortex (FC), there tended to be an Fe repletion effect for higher Fe concentrations (*p* = 0.064).

**Table 2:** Iron concentration and major phospholipid fatty acid composition in different brain regions of male control rats and ID + n-3 FAD rats repleted with an Fe + ALA, ID + ALA, Fe + n-3 FAD, or an ID + n-3 FAD diet for 5 weeks

						***p*** **Value**		
	**Control**	**Fe + ALA**	**ID + ALA**	**Fe + n-3 FAD**	**ID + n-3 FAD**	**Fe**	**ALA**	**Fe × ALA**
Brain Fe*, nmol/g tissue*								
FC	235 ± 11.2	210 ± 8.8	186 ± 10.5*	208 ± 10.5	190 ± 14.7*	0.064	0.97	0.80
OB	300 ± 21.5	303 ± 23.0^‡,¥,Δ^	235 ± 9.3^#^	234 ± 12.1*^,#^	222 ± 18.7*^,#^	0.033	0.009	0.13
Str	263 ± 13.6	239 ± 14.4	223 ± 8.0	237 ± 14.2	204 ± 17.3*	0.12	0.26	0.62
Hip	213 ± 6.7	192 ± 7.0	186 ± 19.8	198 ± 19.4	161 ± 11.9*	0.13	0.97	0.29
Tissue fatty acids, % *of total FA*								
20:5 n-3 (EPA)								
FC	0.05 ± 0.01	0.07 ± 0.01	0.08 ± 0.02	0.07 ± 0.01	0.05 ± 0.01	0.60	0.43	0.23
OB	0.08 ± 0.01	0.10 ± 0.02	0.07 ± 0.01	0.08 ± 0.01	0.07 ± 0.01	0.10	0.61	0.32
Str	0.07 ± 0.01	0.04 ± 0.01	0.07 ± 0.01	0.06 ± 0.01	0.06 ± 0.01	0.27	0.59	0.32
Hip	0.20 ± 0.10	0.11 ± 0.02	0.10 ± 0.02	0.14 ± 0.03	0.09 ± 0.03	0.23	0.72	0.40
22:6 n-3 (DHA)								
FC	13.5 ± 0.2	7.7 ± 0.2*^,‡,¥^	7.7 ± 0.3*^,‡,¥^	3.7 ± 0.1*^,#,Δ^	4.0 ± 0.2*^,#,Δ^	0.51	<0.001	0.53
OB	16.0 ± 0.3	10.1 ± 0.6*^,‡,¥^	9.6 ± 0.4*^,‡,¥^	4.5 ± 0.3*^,#,Δ^	5.2 ± 0.3*^,#,Δ^	0.75	<0.001	0.16
Str	14.1 ± 0.3	7.9 ± 0.3*^,‡,¥^	7.6 ± 0.2*^,‡,¥^	4.5 ± 0.3*^,#,Δ^	4.3 ± 0.3*^,#,Δ^	0.34	<0.001	0.71
Hip	14.5 ± 0.6	9.7 ± 0.6*^,Δ,‡,¥^	7.7 ± 0.2*^,#,‡,¥^	4.2 ± 0.3*^,#,Δ^	4.7 ± 0.5*^,#,Δ^	0.10	<0.001	0.007
Total n-3 FA								
FC	14.0 ± 0.2	8.4 ± 0.3*^,‡,¥^	8.6 ± 0.3*^,‡,¥^	4.0 ± 0.2*^,#,Δ^	4.2 ± 0.2*^,#,Δ^	0.47	<0.001	0.92
OB	16.5 ± 0.3	10.8 ± 0.6*^,‡,¥^	10.2 ± 0.4*^,‡,¥^	4.9 ± 0.3*^,#,Δ^	5.6 ± 0.3*^,#,Δ^	0.89	<0.001	0.13
Str	14.6 ± 0.3	8.5 ± 0.3*^,‡,¥^	8.3 ± 0.2*^,‡,¥^	4.8 ± 0.3*^,#,Δ^	4.7 ± 0.3*^,#,Δ^	0.46	<0.001	0.88
Hip	15.5 ± 1.0	10.6 ± 0.5*^,Δ,‡,¥^	8.7 ± 0.3*^,#,‡,¥^	4.7 ± 0.2*^,#,Δ^	5.2 ± 0.5*^,#,Δ^	0.10	<0.001	0.007

Values are means ± SEM, *n* = 8–10 per group. *Abbreviations*: ALA, alpha-linolenic acid; n-3 FAD, n-3 fatty acid deficient; FC, frontal cortex; Fe, iron; Hip, hippocampus; ID, iron deficient; OB, olfactory bulb; Str, striatum.

*p* Values correspond to two-way ANOVA to test effects of dietary Fe (deficient vs. sufficient) and dietary ALA (deficient vs. sufficient), and Fe × ALA interactions.

*P <0.05 versus control group, analyzed using 1-way ANOVA with diet as between-subject variable followed by post hoc Dunnett’s test to determine whether experimental (repletion) groups differed from age-matched positive-control rats after repletion with Fe and/or ALA.

^#^*P* <0.05 versus Fe + ALA group; ^Δ^*P* <0.05 versus ID + ALA group; ^‡^*P* <0.05 versus Fe + n-3 FAD group; ^¥^P <0.05 versus ID + n-3 FAD group; Analyzed using 1-way ANOVA with diet (excluding control group) as between-subject variable followed by post hoc Tukey’s test to determine differences between experimental (repletion) groups.

### Brain total phospholipid fatty acids

Repleting ID + n-3 FAD rats with ALA significantly increased the relative composition of DHA and total n-3 FA in the total phospholipid fraction of the FC, OB, striatum (Str) and hippocampus (Hip), while no effects were found on EPA (Table 2). Nonetheless, DHA and total n-3 FA composition in all four brain regions remained significantly lower than in controls in all experimental groups. In the Hip, DHA and total n-3 FA were also significantly higher in the groups receiving ALA (Fe + ALA and ID + ALA) compared with the Fe + n-3 FAD and the ID + n-3 FAD groups. However, there was also a significant Fe × ALA interaction, indicating that in the group receiving ALA in combination with Fe (Fe + ALA), the relative composition of DHA and total n-3 FA was significantly higher than in the group receiving ALA alone (ID + ALA) (*p* = 0.011).

### Brain monoamines

At PND 92, dopamine (DA) concentrations concentrations (pmol/mg tissue) in the FC were significantly higher in the ID + n-3 FAD group compared with the age-matched controls (*p* = 0.032), while DA concentrations of Fe + ALA, ID + ALA and Fe + n-3 FAD rats did not differ from controls (*P* >0.05) (Table 3). There was a significant effect of ALA, and an Fe × ALA interaction on serotonin (5-HT) concentrations concentrations in the FC, indicating that the lowering effect of ALA in combination with Fe was attenuated when ALA was provided alone. Fe + ALA rats had significantly higher 5-HT concentrations in the FC than Fe + n-3 FAD rats (*p* = 0.025). A significant Fe × ALA interaction on 5-HT concentrations was also found in the OB (*P* <0.05). 5-HT concentrations in the Fe + n-3 FAD rats were significantly higher than in the ID + n-3 FAD (*p* = 0.039) and Fe + ALA (*p* = 0.049) rats, but this increase was attenuated when Fe was provided in combination with ALA.

**Table 3:** Monoamine concentration (pmol/mg tissue) in three selected brain regions of male control rats and ID + n-3 FAD rats repleted with an Fe + ALA, ID + ALA, Fe + n-3 FAD, or an ID + n-3 FAD diet for 5 weeks

						***p*** **Value**		
***pmol/mg tissue***	**Control**	**Fe + ALA**	**ID + ALA**	**Fe + n-3 FAD**	**ID + n-3 FAD**	**Fe**	**ALA**	**Fe × ALA**
DA								
FC	0.19 ± 0.06	0.40 ± 0.10	0.37 ± 0.09	0.38 ± 0.07	0.55 ± 0.10*	0.46	0.36	0.31
OB	1.28 ± 0.56	0.58 ± 0.07	0.63 ± 0.16	1.02 ± 0.21	0.73 ± 0.19	0.35	0.09	0.30
Str	6.87 ± 1.58	8.25 ± 2.56	9.10 ± 2.53	6.86 ± 2.73	7.68 ± 1.58	0.40	0.74	0.88
DOPAC								
FC	2.22 ± 0.23	3.03 ± 0.50	3.25 ± 0.37	2.28 ± 0.33	3.27 ± 0.50	0.13	0.26	0.31
OB	2.99 ± 0.44	3.35 ± 0.35	2.99 ± 0.25	2.84 ± 0.20	3.51 ± 0.36	0.54	0.88	0.13
Str	12.78 ± 1.00	13.55 ± 0.87	11.97 ± 1.53	13.67 ± 2.37	11.86 ± 0.78	0.64	0.72	0.53
HVA								
FC	1.04 ± 0.12	0.99 ± 0.21	1.29 ± 0.17	1.27 ± 0.21	1.26 ± 0.22	0.44	0.56	0.37
OB	2.22 ± 0.17	1.93 ± 0.19	1.90 ± 0.13	2.12 ± 0.10	2.28 ± 0.25	0.83	0.11	0.66
Str	16.26 ± 1.24	14.69 ± 1.14	14.19 ± 1.13	15.05 ± 1.32	14.42 ± 1.00	0.72	0.82	0.96
5-HT								
FC	3.30 ± 0.66	1.97 ± 0.15^‡^	3.93 ± 0.74	4.67 ± 0.76^#^	3.36 ± 0.63	0.70	0.031	0.005
OB	2.37 ± 0.79	2.29 ± 0.50^‡^	2.76 ± 0.60	4.29 ± 0.55*^,¥,#^	2.22 ± 0.63^‡^	0.10	0.34	0.039
Str	2.58 ± 0.66	2.74 ± 0.46	2.51 ± 0.44	1.77 ± 0.32	2.34 ± 0.52	0.67	0.15	0.38
5-HIAA								
FC	2.31 ± 0.28	2.40 ± 0.29	1.67 ± 0.23	2.28 ± 0.31	2.21 ± 0.31	0.15	0.46	0.23
OB	2.16 ± 0.37	2.15 ± 0.41	2.54 ± 0.21	1.75 ± 0.35	1.73 ± 0.27	0.42	0.16	0.26
Str	2.48 ± 0.51	7.00 ± 1.68	5.10 ± 0.88	4.09 ± 1.21	5.79 ± 1.15	0.68	0.30	0.20
NE								
FC	1.58 ± 0.55	1.92 ± 0.42	1.45 ± 0.43	2.22 ± 0.56	1.40 ± 0.32	0.14	0.76	0.78
OB	2.77 ± 0.80	3.75 ± 0.85	2.27 ± 0.32	2.02 ± 0.29	2.35 ± 0.40	0.54	0.31	0.26
Str	2.70 ± 0.60	3.16 ± 0.55	1.91 ± 0.36	3.25 ± 0.68	2.18 ± 0.39	0.05	0.72	0.71

Values are means ± SEM, *n* = 8–10 per group. *Abbreviations*: ALA, alpha-linolenic acid; DA, dopamine; n-3 FAD, n-3 fatty acid deficient; 5-HIAA, hydroxyindoleacetic acid; 5-HT, serotonin; DOPAC, dihydroxyphenylacetic acid; FC, frontal cortex; Fe, iron; HVA, homovanillic acid; ID, iron deficient; NE, norepinephrine; OB, olfactory bulb; Str, striatum.

*p* Values correspond to two-way ANOVA to test effects of dietary Fe (deficient vs. sufficient) and dietary ALA (deficient vs. sufficient), and Fe × ALA interactions.

*P <0.05 versus control group, analyzed using 1-way ANOVA with diet as between-subject variable followed by post hoc Dunnett’s test to determine whether experimental (repletion) groups differed from age-matched positive-control rats after repletion with Fe and/or ALA.

^#^*P* <0.05 versus Fe + ALA group; ^‡^*P* <0.05 versus Fe + n-3 FAD group; ^¥^P <0.05 versus ID + n-3 FAD group; Analyzed using 1-way ANOVA with diet (excluding control group) as between-subject variable followed by post hoc Tukey’s test to determine differences between experimental (repletion) groups.

### Morris water maze

During the cued task of the water maze, repeated-measures ANOVA revealed a significant effect of trials on distance moved (*p* <0.001) to reach the visible (cued) platform, indicative of learning over the four trials (data not shown). There was no significant effect of diet (*p* = 0.563), nor a significant diet × trial interaction (*p* = 0.671), indicating that sensory and motor deficits did not affect the groups differently. Also, diet did not affect swimming speed across trials in the cued task (*p* = 0.787). However, we did find significant differences in swimming speed between groups during the working memory task. The swimming speed of Fe + ALA rats was significantly higher than of ID + ALA (*p* = 0.042) and ID + n-3 FAD rats (*p* = 0.011). Even though not reaching significance, a similar pattern was also seen during the reference memory task. Therefore, we analyzed working memory and reference memory by using distance moved (in cm) to find the hidden platform and not by escape latency (in s), which could be confounded by between-group differences in swimming speed.

**Figure 1:**
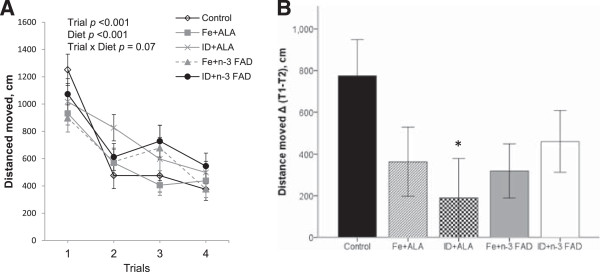
**Working memory task in the Morris water maze (MWM).** Working memory performance in the MWM of male control rats and ID + n-3 FAD rats repleted with an Fe + ALA, ID + ALA, Fe + n-3 FAD, or an ID + n-3 FAD diet for 5 weeks. **(A)** Mean distance moved per trial to reach hidden platform located at different positions across 4 days, with 4 trials per day in the working memory task; **(B)** working memory performance expressed as difference in distance moved between trial 1 and trial 2. Values are means ± SEM, n = 7–8. ALA, alpha-linolenic acid; n-3 FAD, n-3 fatty acid deficient; Fe, iron; ID, iron deficient.

During the working memory task, there was a significant effect of trials and days on mean distance moved (trials: *p* <0.001; days: *p* <0.001) to find the hidden platform (Figure 1A), indicating that overall learning and memory took place across trials and days. There was no significant effect of diet nor a diet × trial interaction. Separate 2 × 2 repeated-measures ANOVA to investigate the effects of Fe and ALA repletion (excluding the control group), revealed a trend towards an effect of Fe repletion for shorter distance moved to find the hidden platform in the working memory task (*p* = 0.061). Furthermore, ID + ALA rats tended to swim a longer distance to reach the hidden platform during trial 2 compared to age-matched controls (*p* = 0.051). Working memory is typically reflected in a rapid reduction of distance moved from trial 1 (when platform position is unknown) to trial 2. Therefore, we calculated the difference in distance moved between trial 1 and trial 2 across all 4 days (Figure 1B). The difference in distance moved between trial 1 and trial 2 was significantly lower in ID + ALA rats compared to age-matched controls (*p* <0.05), indicating impaired working memory.

**Figure 2:**
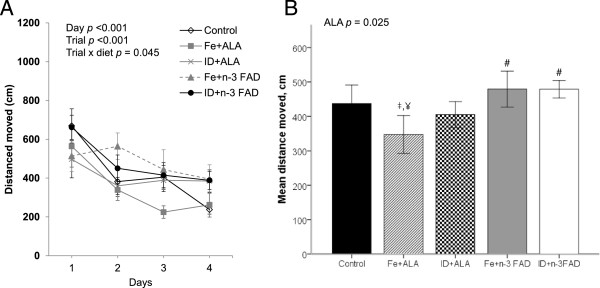
**Reference memory task in the Morris water maze (MWM).** Reference memory performance in the MWM of male control and ID + n-3 FAD rats repleted with an Fe + DHA/EPA, ID + DHA/EPA, Fe + n-3 FAD, or an ID + n-3 FAD diet for 5 weeks. **(A)** Mean distance moved per day to reach hidden platform located at same positions across 4 days, with 4 trials per day in the reference memory task; **(B)** Mean overall distance moved in the reference memory task. Values are means ± SEM, *n* = 7–8. ^#^*P* <0.05 versus Fe + ALA group. ^‡^*P* <0.05 versus Fe + n-3 FAD group. ^¥^P <0.05 versus ID + n-3 FAD group. ALA, alphal-linolenic acid; n-3 FAD, n-3 fatty acid deficient; Fe, iron; ID, iron deficient.

During the reference memory trial, there was a significant effect of days and trials, as well as a significant diet x trial interaction (*p* = 0.045) on distance moved, indicating that not all diet groups showed an improvement in distance moved to find the hidden platform across the 4 trials (Figure 2A). Separate analyses of the distances moved from day 1 to 4 for each diet group revealed that there was no significant improvement in distance moved from day 1 to day 4 (day: *p* >0.05) in the ID + ALA and Fe + n-3 FAD groups, while there was a significant improvement in the Fe + ALA (p <0.001), ID + n-3 FAD (*p* <0.026) and control (*p* <0.001) groups. Furthermore, there was a significant effect of ALA repletion for shorter overall distance moved to find the hidden platform (*p* = 0.025) (Figure 2B). The Fe + ALA group, but not the ID + ALA group, swum a significantly shorter distance to find the hidden platform than the Fe + n-3 FAD (*p* = 0.048) and ID + n-3 FAD (*p* = 0.049) groups.

**Figure 3:**
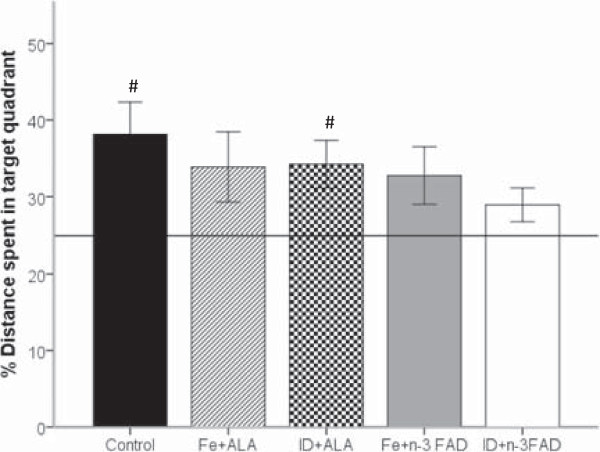
**Probe trial.** Percentage of total distance spent in the target quadrant during the probe trial, when the platform was removed and the rats were allowed to swim freely for 60 s. There were no differences in total distance and time spent in the target quadrant between groups. ^#^Indicates groups that performed above chance level (25%) in the target quadrant; *p* <0.05. Values are means ± SEM, *n* = 7–8. ALA, alpha-linolenic acid; n-3 FAD, n-3 fatty acid deficient; Fe, iron; ID, iron deficient.

After 4 days of reference trials, the platform was removed and the rats were subjected to a probe trial. The percent distance the repletion groups spent in the target quadrant did not significantly differ from controls (Figure 3). However, the ID + n-3 FAD rats tended to spend a shorter distance in the target quadrant than the controls (*p* = 0.077). Only the control and ID + ALA rats showed a distinct preference for the target quadrant, indicated by percent distance spent in target quadrant being significantly above chance level (25%) (Control, *p* = 0.017; ID + ALA, *p* = 0.020).

## Discussion

This is the first study, to our knowledge, to investigate the effects of repleting male adult rats deficient in Fe and n-3 FA with ALA and Fe, alone and in combination, on brain monoamine concentrations and cognitive performance. The experimental model was chosen to reflect the common scenario in low-income countries where children born to mothers with poor n-3 FA status consume a diet low in Fe and n-3 FA throughout childhood and only begin to consume a diet sufficient in one or both nutrients in early adulthood as dietary quality and variety improves.

The provision of ALA to ID + n-3 FAD rats for 5 weeks (PND 56–91) significantly increased the relative composition of DHA in total phospholipid FA of different brain regions to ~ 50 - 70% of control values. In contrast, the provision of Fe resulted in near complete recovery of brain Fe (~90–100% of controls). In the Hip, the synergistic Fe × ALA interaction for higher DHA levels may be explained by Fe being a co-factor of hepatic desaturases and elongases, which are responsible for the conversion of ALA to EPA and DHA [26,27]. Thus, providing ALA in combination with Fe to double-deficient rats might enhance the conversion of ALA into its respective long-chain derivative DHA. Whether this finding can be translated to the human situation is not clear, as previous studies have shown that, in contrast to humans, the conversion of ALA to EPA and DHA is more efficient in rats [19,20]. Nevertheless, Smuts et al. showed that providing ID school children with iron-fortified soup not only improved Fe but also n-3 FA status [18].

Rats that remained ID during the depletion period showed a ~15% reduction in food intake, which was also reflected by ~15% lower body weight compared to the groups repleted with Fe. This is consistent with several previous studies reporting decreased food intake associated with poor growth in rats suffering from ID anaemia [12,28-30]. However, previous depletion studies showed that severe ID decreased relative weight gain (g weight gain per g food intake) [12,28], while in the current study, relative weight gain was the same in rats repleted with Fe and remaining ID. Since neurotransmitters, including DA and 5-HT, are involved in the regulation of food intake [31], it is possible that the reduced food intake of ID rats is related to alterations in monoaminergic neurotransmission. Also, the reduced body weight in ID rats may explain why swimming speed in the MWM was lower in ID + ALA and ID + n-3 FAD rats.

We previously showed that the provision of an ID + n-3 FAD diet for 5 weeks post-weaning resulted in an additive 1- to 2-fold increase in DA concentrations in the OB and Str, and decreased 5-HT concentrations relative to controls in the OB at PND 56 [12]. In the current study, at PND 91, rats that remained ID + n-3 FAD had significantly higher DA concentrations than controls (+189%) in the FC. In contrast, DA concentrations in rats that received ALA and Fe, alone or in combination, did not differ from controls, consistent with our previous study in which DHA/EPA was provided alone and in combination with Fe [13]. However, in contrast to our previous study, where we found a significant Fe × DHA/EPA interaction on the DA metabolite dihydroxyphenylacetic acid (DOPAC) in the OB and Str [13], we found no Fe × ALA interactions on DOPAC in the current study. However, we did find significant antagonistic Fe × ALA interactions on 5-HT in FC and OB, which suggest that the provision of Fe in combination with ALA to double-deficient rats affects 5-HT concentrations differently from the provision of Fe and ALA alone. The FC is one of the last brain areas to become fully mature and previous studies have reported dramatic increases in DA- and 5-HT-mediated neurotransmission in the FC during adolescence [32-34]. This may explain why most effects of Fe and ALA repletion and of continued ID + n-3 FAD depletion at PND 91 were observed in the FC. Several rodent studies have previously investigated whether alterations in monoaminergic neurotransmission caused by ID can be reversed by Fe repletion [35-40]. These studies varied in timing and severity of ID, in timing and dose of iron repletion, as well as in the brain regions observed. Generally, the effects of ID induced during gestation and pre-weaning have been shown to be persistent [35,36,39,40]. Rodent studies using a model of post-weaning ID are limited. To our knowledge, to date, only one study has shown that alterations in extracellular DA in the caudate putamen caused by ID introduced post-weaning were mostly reversible by iron repletion [41]. Also, few studies have investigated whether the detrimental effects of n-3 FAD (induced over two generations) on monoaminergic neurotransmission can be reversed with ALA repletion [42,43]. Kodas et al. investigated synaptic levels of DA and 5-HT in basal conditions and after pharmacological stimulation in rats that were n-3 FA depleted and in rats that were repleted with an ALA sufficient diet starting at different time points (birth, PND 7, PND 14 or PND 21) [42,43]. N-3 FA deficiency altered DA and 5-HT release in basal conditions and under stimulation in the rat Hip. The provision of ALA during the first two weeks of postnatal life reversed these alterations, while they persisted in rats that received the repletion diet only from PND 21.

Nonetheless, our results cannot be directly compared with the results of these studies, as the experimental rats in our study were deficient in both Fe and n-3 FA before receiving the repletion diets. Thus, the effects of Fe or n-3 FA repletion could have been confounded by the untreated deficiency. It is well known that neurotransmitter systems can adapt to chronic stressors, such as drug and alcohol exposure [44]. This could explain why certain monoamines that were altered in the ID + n-3 FAD rats after the depletion study (PND 56) were no longer altered in the rats that remained ID + n-3 FAD during the repletion study (at PND 92). We speculate that providing double-deficient rats with one nutrient only affects the adapted system differently than the provision of both nutrients, e.g. by re-activating different mechanisms, which may explain the different effects on brain 5-HT concentrations when Fe and ALA were provided alone and in combination.

Disturbances in DA and 5-HT neurotransmission can impair learning and memory [45]. The results from the MWM testing indicate that the provision of ALA alone to double-deficient rats significantly impairs working memory performance compared to age-matched controls. This finding is consistent with our previous study, where the provision of DHA/EPA alone also produced significant deficits in working memory performance compared to age-matched controls [13]. The mechanism of this effect is uncertain. Spatial working memory is mainly hippocampal-dependent but is sub-served in part by the prefrontal cortex [45,46]. Therefore, the working memory results might be explained by the differential effects of ALA on 5-HT in the FC when provided alone and in combination with Fe. There is evidence suggesting that 5-HT is involved in working and reference memory processes [45,47]. Unfortunately, data on monoamine concentrations in the Hip are not available in the current study.

We consistently demonstrated that rats repleted with ALA or Fe alone showed no learning effect in the reference memory task, indicated by a lack of improvement in distance swum across trials and days to find the hidden platform. On the other hand, rats that received ALA in combination with Fe exhibited a marked improvement. This finding could again be attributed to the differential effects of ALA and Fe on 5-HT concentrations in the FC when provided alone or in combination. Consistently, a recent study reported that ID rats fed with Fe and a mixture of essential FA, containing equal amounts of LA and ALA, exhibited improved learning and memory performance in the MWM compared with controls, while the rats fed with Fe alone performed worse [48]. In contrast to our previous study that provided DHA/EPA, alone and in combination [13], we did find that the provision of ALA significantly shortened overall distance moved to find the hidden platform during the reference memory task. However, this beneficial effect of ALA was only apparent in the Fe + ALA group, which swam a significantly shorter distance to find the platform than the groups that remained double-deficient or received Fe or ALA only. Since spatial learning and memory tested in the MWM is mainly hippocampus-dependent [49,50], the higher relative composition of DHA in total phospholipid FA in the Hip of rats that were fed ALA in combination with Fe might explain the improved reverence memory performance in this group. Nevertheless, it may be that ALA exerted beneficial effects on cognitive performance independent of its conversion to EPA and DHA.

Since spatial working and reference memory is mainly hippocampal-dependent, it is a limitation of this study that monoamine concentrations were not analyzed in the Hip. Also, the assessment of other neurotransmitters, such as glutamate and gamma-aminobutyric acid, or other factors involved in learning and behaviour, such as myelination, neuronal inflammation, and processes of morphogenesis and cell growth, would have been valuable.

## Conclusion

Consistent with our previous study in which a mixture of DHA/EPA was provided, alone and in combination with Fe, feeding either ALA or Fe alone to adult rats with both ID and n-3 FAD affected brain 5-HT concentrations differently from the provision of ALA and Fe in combination, and even exacerbated the working memory deficits associated with combined deficiency. However, in contrast to our previous study, the provision of ALA had a beneficial effect on reference memory, particularly when provided in combination with Fe, which may be explained by the enhancing effect of Fe on the conversion of ALA to DHA in the Hip. These findings are of relevance to human populations, as many children from a low-socioeconomic background may suffer from both ID and n-3 FAD due to poor quality diets. Furthermore, in populations with a low fish intake, improving DHA and EPA status by increasing the consumption of ALA-rich oils (e.g. flaxseed, canola or soybean oils) might be a more suitable approach. Our results indicate that in such populations, it may be crucial to provide ALA-rich oils and foods in combination with Fe.

## Methods

### Rats and diets

**Figure 4:**
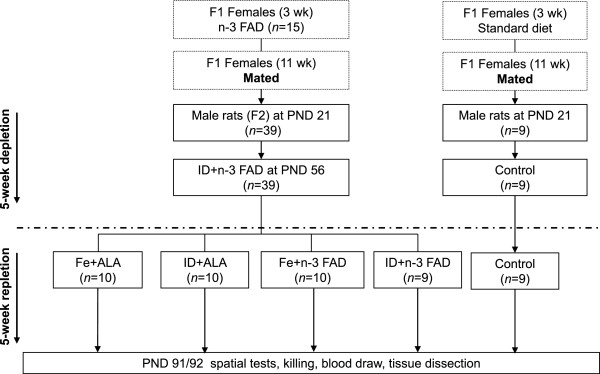
**Flow diagram of the experimental design.** n indicates the numbers in each experimental group. Boxes in dashes indicate that these parts of the experiment were done at a commercial animal breeding center. ALA, alpha-linolenic acid; n-3 FAD, n-3 fatty acid deficient; Fe, iron; ID, iron deficient.

All experimental procedures were approved by the Veterinary Office of the Department of Health of the Canton of Zürich. Male Wistar rats were first made n-3 FAD over two generations. Male rats were chosen to avoid potential confounding effects of estrogen that have been reported in previous studies of ID and n-3 FAD [51,52]. At a commercial animal breeder (RCC), female Wistar rats (PND 21) were fed an n-3 FAD diet (detailed below), and were mated at 11 weeks with 12-week-old male breeders of the same strain. After the mating period, the females continued to consume the n-3 FAD diet and the rat pups were kept with their dams until weaning. After weaning at PND 21, male n-3 FAD rats received a concurrent ID and n-3 FAD (ID + n-3 FAD) diet for a depletion period of 5 weeks. At PND 56, the now ID + n-3 FAD rats (*n* = 39) were randomly divided into four groups (Figure 4). The repletion study was designed as a 2 × 2 factorial experiment and the four groups received the following diets for a period of 5 wk: 1) a continued ID and n-3 FAD (ID + n-3 FAD, *n* = 10); 2) Fe replete and n-3 FAD (Fe + n-3 FAD, *n* = 10); 3) Fe and ALA replete (Fe + ALA, *n* = 10); or 4) ID and ALA replete (ID + ALA, *n* = 10) diet. Furthermore, we included an age-matched (positive-) control group from dams that received the n-3 FA and Fe sufficient basal AIN-93G diet.

The purified experimental diets were obtained commercially (Dyets, Bethlehem, PA, USA) and were based on the AIN-93G [53] formulation with modifications in Fe content and fat source (Table 4). All diets contained 10% fat. The n-3 FAD diets contained hydrogenated coconut oil at 81 g/kg diet, and safflower oil at 19 g/kg diet. The ALA repletion diets contained hydrogenated coconut oil at 60 g/kg diet, safflower oil at 32 g/kg diet and flaxseed oil at 8 g/kg diet. All diets included n-6 FA in the form of LA. The Fe replete diets contained 35 mg Fe/kg, and the ID diets during the preceding depletion period of 5 weeks contained 3 mg Fe/kg. For the repletion period, the Fe dose in the ID diets was increased to 10 mg Fe/kg diet in order to avoid potential deaths from prolonged severe ID anemia. All diets were custom prepared in powdered form to avoid lipid oxidation during pelletization, vacuum packed in bags of 2 kg, and stored at -20°C until use.

**Table 4:** Composition of experimental diets during depletion period^*^

**Ingredient**	**g/kg diet**		
Casein, vitamin free	200		
Carbohydrate	600		
Cornstarch	368		
Sucrose	100		
Deytrose	132		
Cellulose	50		
Mineral mix^#^	35		
Vitamin mix	10		
L-Cystein	3		
Choline bitartrate	2.5		
TBHQ	0.02		
Fat	100		
	**n-3 FAD**^**†**^	**ALA sufficient**^**‡**^	**Control**^**¥**^
Coconut oil (hydrogenated)	81.0	60.0	30.0
Soybean oil	0.0	0.0	70.0
Safflower oil	19.0	32.0	0.0
Flaxseed oil	0.0	8.0	0.0

^*^Experimental diets were based on the AIN-93G formulation with modifications in iron content and fat source.

^#^The mineral mix provided 35 mg iron/kg diet in the iron sufficient diets and 3 mg iron/kg diet in the iron deficient diets. n-3 FAD, n-3 fatty acid deficient; ID, iron deficient; TBHQ, t-butylhydroquinone.

^†^Providing approximately 15.1 g/kg diet linoleic acid (LA) and 0.04 g/kg diet α-linolenic acid (ALA).

^‡^Providing approximately 20.0 g/kg diet LA and 46.0 g/kg diet ALA.

^¥^AIN-93G formulation providing approximately 35.7 g/kg diet LA and 4.8 g/kg diet ALA.

### Tissue collection

At the end of the repletion period (PND 91 and 92) rats were exposed to CO_2_ to introduce unconsciousness for blood collection by cardiac puncture and then killed by decapitation. The brains were rapidly removed and OB, FC, Str and Hip were isolated from both hemispheres by freehand dissection. Brain regions were placed into pre-weighed Eppendorf tubes, their wet weight was recorded and then they were snap-frozen in liquid nitrogen and stored at -80°C. Per brain area, tissues obtained from the left and right hemispheres were alternately used for the analysis of brain iron and FA content.

### Brain iron analysis

Brain regions were homogenized and digested with nitric acid according to Erikson et al. [54], and total Fe concentrations measured using graphite furnace atomic absorption spectrometry (AAS; Perkin Elmer AA400, Beaconsfield, Bucks, UK) as described previously [12].

### Total phospholipid fatty acid analysis

Lipids were extracted from each brain region with chloroform:methanol (2:1 v/v) by a modification of the method of Folch et al. [55]. The lipid extracts were concentrated and the neutral lipids separated from the phospholipids by thin-layer chromatography (TLC) (Silica gel 60 plates, 10 x 20 cm, Merck, Darmstadt, Germany) and eluted with diethyl ether: petroleum ether: acetic acid (30:90:1 v/v/v). The lipid band containing phospholipids was removed from the TLC plate and transmethylated with methanol:sulphuric acid (95:5 v/v) at 70°C for 2 h to yield fatty acid methyl esters (FAME). The resulting FAME was extracted with water and hexane. The organic layer was evaporated, re-dissolved in hexane and analyzed by QP GC-EI-MS on an Agilent Technologies 7890A GC system equipped with an Agilent Technologies 5975C VL mass selective detector. The GC separation of FAME was carried out on an HB-5MS capillary column (30 m × 0.250 mm × 0.25 μm; Agilent J&W) using helium as the carrier gas at a flow rate of 0.9 mL/min. The GC injector was held at a temperature of 250°C and the MS source and QP were maintained at temperatures of 230°C and 150°C respectively. The injection volume of the sample solution was 1 μL, using a split ratio of 1:25 for the brain samples. The oven temperature started at 140°C and was programmed at +3°C/min from 140 to 220°C, held at 220°C for 2 min, then programmed at +3°C/min to 230°C and held at 230°C for 10 min. Total analysis time was 45 min. Mass spectrometry with 70 eV EI was carried out in full scan acquisition mode and all mass spectra were acquired over the *m*/*z* range of 50–750. Quantification of FAME was done using the selected ion extraction (SIE) method.

For the calibration of FAME, a standard reference mixture of 26 FAME (Nu-Check-Prep, Elysian, MN) and three single FAME standards (Larodan Fine Chemicals AB, Malmö, SE) were injected at equal concentrations ranging between 1 and 400 ng in 1 μL and calibration plots for each FAME were obtained using peak areas from mass chromatograms, using C17:0 as an external standard. Data analysis was performed using MSD ChemStation software (Agilent G1701EA version E.02.00.493). Relative percentages of FA were calculated by taking the concentration of a given FA derivative as a percentage of the total concentration of all FA identified in the sample.

### Brain monoamine analysis

DA dihydroxyphenylacetic acid (DOPAC), homovanillic acid (HVA), 5-HT, 5-hydroxyindoleacetic acid (5-HIAA) and norepinephrine (NE) were measured in Str, FC and OB using reverse-phase HPLC with electrochemical detection. Several previous rodent studies have shown that monoaminergic neurotransmission in these brain regions was affected by ID and/or n-3 FAD [35,36,43,56-58]. We initially also planned to measure monoamine concentrations in the Hip. However, unfortunately the Hip samples got lost during shipment to the analytical laboratory. The regions were prepared and analyzed as described elsewhere with minimal modifications [7,41]. Briefly, 1 g of tissue was diluted into 10 mL PBS spiked with complete protease inhibitor tablets (Roche Diagnostics, Mannheim, Germany). Samples were homogenized on ice with a PT 1200E Polytron tissue homogenizer using acid washed plastic dispersing-aggregates (single use; Kinematic AG, Littau, Switzerland). Homogenates were then passed 10 times through a 1 mL insulin syringe with 29G × 12.7 mm needle (Beckton Dickinson AG, Allschwil, Switzerland) to further homogenize the tissue. Homogenates were sonicated with 5 up and down strokes. 50 μL of the homogenate were added to 50 μL 0.24 M HClO4. 10 μL internal standard (dihydroxybenzylamine, 1.1 mg/L) was added to track efficiency of extraction in all samples. The spiked solution was sonicated for 10 s, filtered using Costar Spin-X filter tubes 0.2 μm (Corning, NY, USA), and 10 μL was injected onto the HPLC by an ESA 542 refrigerated auto sampler, as described by Bianco et al. [7].

### Morris water maze test

The MWM was used to evaluate spatial learning and memory performance. The MWM test was carried out in the dark phase of the cycle in eight randomly selected rats from the experimental groups and from the control group. The maze consisted of a black, circular, fiberglass tank (2 m in diameter) that was positioned in the middle of a testing room enriched with unique distal spatial cues under dim lighting condition (12 lux as measured from the maze centre). The tank was filled daily with fresh tap water (22 ± 1°C) to a depth of 30 cm. A black, circular platform (11 cm in diameter) submerged 1.5 cm below the water surface was used as an escape platform invisible to the rats. All trials were monitored by a video camera installed above the water maze, and analyzed using Ethovision software version 3.1 (Noldus).

The rats underwent four phases of testing in the water maze on 10 consecutive days: 1) Cued task (1 d, PND 80); 2) Working memory task (4 d, PND 81–84); 3) Reference memory task (4 d, PND 85–88) and; 4) Probe test (1 d, PND 89).

First, the rats were familiarized to the water maze procedure in a cued task, which also served as a test for non-specific sensory motor differences. In this pre-training phase, the platform was positioned in the center of the maze and was made visible with a local cue (flag). Each rat underwent 4 consecutive trials, with the starting position randomly selected from four release points (N, W, S, and E). Each rat was released into the water facing the outer edge of the pool and was allowed a maximum of 90 s to escape onto the platform. If a rat failed to do so, a maximum escape latency of 60 s was recorded, and the rat was guided to the platform by the experimenter and allowed to stay on it for an inter-trial interval of 15 s before the next trial.

The next day, in the working memory task, the platform position (randomized across rats) varied from one day to the next in a non-repetitive manner, but remained submerged, but without the local cue, at the same position across the four trials within a day. The four platform positions were 16 cm off the wall in the NW, NE, SE and SW directions of the tank. The starting positions varied randomly across the trials amongst the four possible release points (N, W, S and E). The rats were allowed 90 s to find the platform and the inter-trial interval was 15 s. This task taxes the flexible use of day-dependent short-term (working) memory [59]. As previously, when a rat failed to locate the platform within 90 s, it was guided to it and a maximum escape latency of 60 s was recorded.

The reference memory task began the day after completion of the working memory task. This task assessed long-term spatial memory, by applying a procedure in which the platform remained in a constant location across trials (4 trials per day) and across training days (4 days), while the starting positions changed randomly across trials. As described above, the rats were allowed to search for the platform for 90 s, with an inter-trial interval of 15 s.

A probe test was conducted 24 h after the final reference trial to assess memory retention. To this end, the platform was removed from the tank and the rats were released in the quadrant opposite to the trained platform location. The rats were allowed to swim freely in the water maze for 60 s (one trial only). The distance covered in each quadrant was recorded.

After each training day in the water maze, the rats were dried under a red-light heat lamp, before being returned to their home cages.

### Statistical analysis

Data were analyzed using IBM SPSS Statistics software (version 20.0). Data were examined for normality of distribution by a Shapiro-Wilk test and the presence of outliers (±3 SD from the mean, boxplots). Homogeneity of variance was examined by Levene’s test. Datasets that significantly deviated from normality and/or variance homogeneity were transformed prior to interferential statistical analysis using ANOVA.This repletion study was designed as a 2 × 2 factorial experiment. Figure 1 shows that the control group did not have the same dietary history as the repletion groups. The control group was used as a positive-control to examine whether repletion with either nutrient alone or in combination can reverse deficits in cognition and changes in biochemical indicators associated with double-deficiency to a level of age-matched rats that received an n-3 FA and Fe sufficient basal AIN-93G diet throughout their lives. Therefore, we performed two different statistical analyses with the following aims: 1) 2-way ANOVA for biochemical indicators and 2-way repeated measures ANOVA (with trials and days as repeated measurement variables) for MWM data, excluding the control group, to determine the effects of Fe (sufficient vs. deficient) and ALA (sufficient vs. deficient) repletion and their interactions in double-deficient rats. Tukey’s tests (Fisher’s least significant difference tests for MWM data) were used for appropriate pair-wise comparison following the emergence of significant effects from the overall ANOVA. Significant treatment effects in the absence of a significant interaction effect indicated additive effects of the treatments, whereas a significant interaction implied synergism or antagonism; 2) 1-way ANOVA with diet as between-subject variable followed by post hoc Dunnett’s test to determine whether experimental (repletion) groups differed from age-matched positive-control rats after repletion with Fe and/or ALA.

For the MWM probe trial, the percentage distance and time spent in the target quadrant were compared between groups using 1-way ANOVA with diet as between-subject variable followed by Fisher’s least significant difference tests. Furthermore, one-sample t-tests were carried out to investigate whether distance moved in the target quadrant was above the chance level of 25%. The results were expressed as means ± SEM and differences were considered significant at *p* <0.05.

## Abbreviations

ALA: Alpha-linolenic acid; ARA: Arachidonic acid; DA: Dopamine; DHA: Docosahexaenoic acid; DOPAC: Dihydroxyphenylacetic acid; EPA: Eicosapentaenoic acid; n-3 FA: n-3 fatty acids; n-3 FAD: n-3 fatty acid deficiency; FAME: Fatty acid methyl ester; FC: Frontal cortex; Fe: Iron; 5-HIAA: 5-hydroxyindoleacetic acid; Hip: Hippocampus; 5-HT: Serotonin; ID: iron deficiency; LA: Linoleic acid; MWM: Morris water maze; OB: Olfactory bulb; PND: Postnatal day; PUFA: Polyunsaturated fatty acid; Str: Striatum; TLC: Thin-layer chromatography.

## Competing interests

The authors declare that they have no competing interests.

## Authors’ contributions

The authors’ responsibilities were as follows: MBZ, CMS and JB designed research; JB conducted research; JB analyzed data; JB prepared the first draft of the paper that was revised by CMS and MBZ, and JB had primary responsibility for final content. All authors read and approved the final manuscript.
